# Transmission of Infection from Rats to Man

**Published:** 1900-09

**Authors:** 


### Abstracts and Selections.

### Transmission of Infection From Rats to Man.

If the manner in which rats contract plague is to a great extent uncertain, it is no less uncertain how the disease is usually transmitted to man by these animals. Direct transmission from this animal to man by means of a bite from an infected animal must be extremely rare. Only two instances are given in which such an occurence has been observed. In one the patient had been bitten by a rat on both great toes; the skin was penetrated, and blood oozed from the wounds; an attack of plague followed, from which he ultimately recovered. In the second instance gangrene developed around the wound, and the patient died.

There is very good reason to believe that plague may be and is spread from rats to other rats, and perhaps to human beings by insects, of which probably the flea is the most important.—*Maryland Medical Journal.*

